# GLUTATHIONE DEFICIENCY AS A NOVEL RISK FACTOR FOR ALZHEIMER’S DISEASE: IMPLICATIONS FOR GLYNAC SUPPLEMENTATION

**DOI:** 10.1093/geroni/igae098.0810

**Published:** 2024-12-31

**Authors:** George Taffet, Premranjan Kumar, Ob Osahon, Chimaoge Onyechi, Charles Minard, Joseph Masdeu, Rajagopal Sekhar

**Affiliations:** Houston Methodist Hospital, Houston, Texas, United States; Baylor College of Medicine, Houston, Texas, United States; Baylor College of Medicine, Houston, Texas, United States; Nantz National Alzheimer’s Center, Houston, Texas, United States; Baylor College of Medicine, Houston, Texas, United States; Nantz National Alzheimer’s Center, Houston, Texas, United States; Baylor College of Medicine, Houston, Texas, United States

## Abstract

Cognitive impairment in Alzheimer’s disease (AD) is linked to defects such as oxidative stress (OxS), mitochondrial dysfunction, inflammation and insulin resistance. These defects also occur in aging where they are linked to deficiency of the intracellular antioxidant glutathione. We recently reported that GSH deficiency was present in the brains of cognitively-impaired aged mice, and that both glutathione deficiency and cognition improved after supplementation with GlyNAC (combination of glutathione precursors glycine and N-acetylcysteine). We hypothesized that patients with AD with mild cognitive impairment (MCI) would have greater glutathione deficiency than older-adults (OA), and tested our hypothesis by comparing intracellular red-blood cell glutathione concentrations in young-adults (YA), OA without cognitive impairment, and patients with AD+MCI (AD-MCI). Red-blood cell total glutathione (tGSH), reduced GSH (rGSH) and oxidized glutathione (GSSG) concentrations from 32 AD-MCI patients in an ongoing randomized clinical trial were compared to historical data from 32 OA and 19 YA from our published studies to determine whether, and to what extent glutathione levels are deficient in AD-MCI. We found that YA had the highest levels of tGSH and rGSH, followed by OA and the lowest levels were seen in AD-MCI. GSSG levels were also significantly lower in AD-MCI compared to OA and YA. These data indicate that AD-MCI is associated with severe glutathione deficiency. Because GlyNAC improves/corrects glutathione deficiency, (a potential key defect in AD), a randomized clinical trial is in progress to test whether GlyNAC supplementation can improve glutathione deficiency, metabolic and mitochondrial defects, and cognitive function in patients with AD-MCI.

